# Factors associated with presenting >12 hours after symptom onset of acute myocardial infarction among Veteran men

**DOI:** 10.1186/1471-2261-12-82

**Published:** 2012-09-28

**Authors:** Kelly McDermott, Charles Maynard, Ranak Trivedi, Elliott Lowy, Stephan Fihn

**Affiliations:** 1 Osher Center for Integrative Medicine, University of California, San Francisco, 1545 Divisadero St., 3rd Floor, Box 1726, San Francisco, CA 94115, USA; 2 Health Services Research & Development Northwest Center of Excellence, Veterans Affairs Puget Sound Health Care System, 1100 Olive Way, Suite 1400, Met Park West, Seattle, WA, 98101, USA

**Keywords:** Veterans, Acute myocardial infarction, Time from symptom onset, Delayed presentation

## Abstract

**Background:**

Approximately 2/3 of Veterans admitting to Veterans Health Administration (VHA) facilities present >12 hours after symptom onset of acute myocardial infarction (AMI) (“late presenters”). Veterans admitted to VHA facilities with AMI may delay hospital presentation for different reasons compared to their general population counter parts. Despite the large descriptive literature on factors associated with delayed presentation in the general population, the literature describing these factors among the Veteran AMI population is limited. The purpose of this analysis is to identify predictors of late presentation in the Veteran population presenting with AMI to VHA facilities. Identifying predictors will help inform and target interventions for Veterans at a high risk of late presentation.

**Methods:**

In our cross-sectional study, we analyzed a cohort of 335 male Veterans from nine VHA facilities with physician diagnosed AMI between April 2005 and December 2006. We compared demographics, presentation characteristics, medical history, perceptions of health, and access to health care between early and late presenting Veterans. We used standard descriptive statistics for bivariate comparisons and multivariate logistic regression to identify independent predictors of late presentation.

**Results:**

Our cohort was an average of 64 ± 10 years old and was 88% white. Sixty-eight percent of our cohort were late presenters. Bivariate comparisons found that fewer late presenters had attended at least some college or vocational school (late 53% vs. early 66%, p = 0.02). Multivariate analysis showed that presentation with ST-elevation myocardial infarction (STEMI) was associated with early presentation (OR = 0.4 95%CI [0.2, 0.9]) and ≥2 angina episodes in the prior 24 hours (versus 0-1 episode) was associated with late presentation (OR = 7.5 95%CI [3.6,15.6]).

**Conclusions:**

A significant majority of Veterans presenting to VHA facilities with AMI were late presenters. We found few differences between early and late presenters. Having a STEMI was independently associated with early presentation and reporting ≥2 angina episodes in the 24 hours prior to hospital admission was independently associated with late presentation. These independent predictors of early and late presentation are similar to what has been reported for the general population. Despite these similarities to the general population, there may be untapped opportunities for patient education within the VHA to decrease late presentation.

## Background

While many institutions have successfully decreased time from hospital presentation to treatment for acute myocardial infarction (AMI), patient delay from symptom onset to hospital presentation has not decreased [1-3]. Eighteen percent of Medicare beneficiaries with AMI present >12 hours after symptom onset [4]. These patients are more likely to have irreversible myocardial damage and less likely to receive reperfusion therapy [5-7]. Approximately 2/3 of Veterans presenting to Veterans Health Administration (VHA) facilities with AMI present more than 12 hours after symptom onset (“late presenters”) [8,9]. Veteran patients with AMI differ from their general population counter parts in a number of ways including increased mental and physical comorbidity and disability [10]. Based on underlying population differences, predictors of late presentation in the Veteran population may be different compared to those reported for the general population. To the best of our knowledge, there have been no quantitative assessments of late presentation among Veterans. The purpose of this analysis is to identify predictors of late presentation in the Veteran population so as to inform and target interventions to decrease late presentation at VHA facilities.

## Methods

This is a cross-sectional study to determine factors related to time from symptom onset to hospital presentation for Veterans with AMI admitted to a national sample of VHA facilities. We conducted a secondary data analysis of Veterans enrolled in the VHA Acute Coronary Syndrome (ACS) Study, a prospective study of patients who had physician confirmed ACS and were admitted to one of the nine participating VHA facilities between April 2005 and December 2006 [9]. The VHA ACS Study sites included: four percutaneous coronary intervention (PCI) capable facilities (located in Portland OR, Denver CO, Minneapolis MN, and Durham NC); the four geographically closest and highest referring non-PCI capable facilities (located in Roseburg OR, Sheridan CO, Fargo ND, and Salisbury NC); and, one PCI capable facility to increase recruitment (located in Tampa FL). The VHA ACS Study was approved by all local Institutional Review Boards. The current study is an approved secondary analysis using deidentified ACS Study data.

### Study population

**Figure 1:**
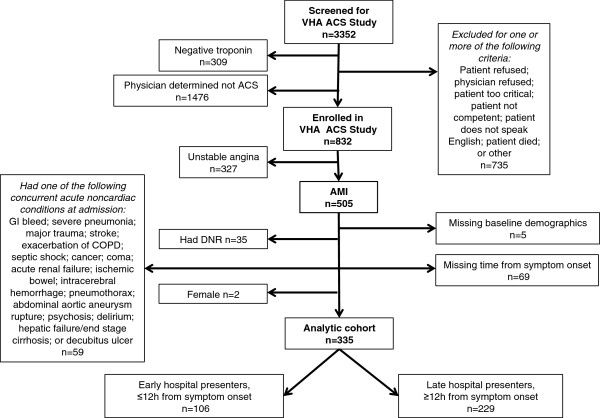
**Study flow diagram ofVeterans excluded from theVHA ACS Study andanalytic cohort.**

Our analytic cohort consisted of 335 Veterans who presented to VHA facilities with AMI and were enrolled in the VHA ACS Study. AMI was diagnosed by elevated troponin and electrocardiogram (ECG) evidence and was confirmed by a physician. (Figure 1). For analytic purposes, we excluded enrollees missing demographic data (n = 5) or time from symptom onset (n = 69). We also excluded the sickest Veterans based on atypical factors associated with presentation. These excluded Veterans had *do not resuscitate* (DNR) in their medical record (n = 35) or presented with one of the following concurrent acute noncardiac conditions: gastrointestinal bleed, severe pneumonia, major trauma or fracture, stroke, exacerbation of chronic obstructive pulmonary disease (COPD), septic shock, cancer, coma, acute renal failure, ischemic bowel, intracerebral hemorrhage, pneumothorax, abdominal aortic aneurysm rupture, psychosis, delirium, hepatic failure/end-state cirrhosis, or decubitus ulcer (n = 59). Two female Veterans were also excluded based on their gender. Table 1 compares Veterans included in and excluded from our analytic cohort. Excluded Veterans were more likely to come in early, were significantly older and significantly more likely to have congestive heart failure (CHF) or diabetes. (Table 1).

**Table 1:** **Comparison of Veterans excludedfrom analytic cohort basedon missing data, havingconcurrent noncardiac conditions ordo not resuscitate orderat presentation**

	**Included n = 335**	**Excluded n = 170**	***P***
	***%(n)***	***%(n)***	
Late presentation*	68.4 (231)	30.6 (50)	<0.01
**Demographics**			
Age	64.4 ±10.5 (335)	68 ±11.1 (163)	<0.01
Male	100 (335)	95.3 (162)	0.01
Lives alone	29.6 (99)	27.1 (46)	0.56
Attended some college/vocational	57 (191)	40 (68)	<0.01
Miles from home zip to VHA facility	48.4 ±123 (199)	47.5 ±71.8 (79)	0.96
Employment status			<0.01
Full time	21.4 (67)	10.1 (15)	
Part time	13.7 (43)	8.1 (12)	
Not employed	64.9 (203)	81.8 (121)	
Family history of CAD	47.9 (146)	50 (70)	0.91
**Presentation characteristics**			
STEMI	18.2 (61)	11.2 (19)	0.04
Angina episodes in previous 24 h			<0.01
0-1	38.5 (129)	54.7 (93)	
≥2	30.1 (101)	12.4 (21)	
Missing	31.3 (105)	32.9 (56)	
Tertiary facility	90.1 (302)	80.6 (137)	0.03
**Medical History**			
≥1 angina episode per day over prior 4 weeks	24.2 (81)	20.6 (35)	0.37
Cardiac procedure (PCI or CABG)	39.4 (132)	45.3 (77)	0.2
Prior myocardial infarction	38.2 (128)	38.2 (65)	0.99
Prior congestive heart failure	16.1 (54)	30.6 (52)	<0.01
Prior stroke	12.8 (43)	12.9 (22)	0.97
Diabetes	41.5 (139)	51.8 (88)	0.03
PTSD	12.2 (41)	8.8 (15)	0.25
Alcohol or substance abuse	20.2 (67)	17.6 (30)	0.53

* Late presentation is hospital admission >12 hours after symptom onset of acute myocardial infarction.

*CAD* coronary artery disease; *STEMI* ST elevation myocardial infarction; *PCI* percutaneous coronary intervention; *CABG* coronary artery bypass grafting; *PTSD* post traumatic stress disorder.

### Measurement and study variables

Demographic and medical history data were abstracted from patient medical records at the time of enrollment by trained local study coordinators. Other data were collected in patient interviews and with self administered questionnaires. The outcome of interest was late presentation, which we defined as presentation to a VHA facility >12 hours after the self reported time of initial symptom onset. We compared early and late presenting Veterans based on demographics including age, race, marital status, whether they lived alone, education, distance traveled, employment status, and a family history of coronary artery disease (“Do you have a parent or sibling who suffered from heart disease before the age of 55 if male and 65 if female?”). We compared hospital presentation characteristics including the number of angina episodes the Veteran had in the 24 hours prior to admission, and whether the Veteran had a discharge diagnosis of ST elevation myocardial infarction (STEMI) for this admission. We also compared whether the facility was PCI capable (tertiary status). Angina frequency over the prior 4 weeks was self reported but all of the other medical history variables were identified in medical chart abstraction. These include indicators of any prior invasive cardiac procedures (i.e. PCI or coronary artery bypass grafting (CABG)), a prior MI, CHF, prior stroke, diabetes, post traumatic stress disorder (PTSD) and alcohol or substance abuse. We indicated the Veteran’s perception of his health based on responses to “How often do you think or worry that you may have a heart attack or die suddenly?” and access to care based on the ranking of difficulty in response to the question “Overall, how difficult is it for you to get medical care when needed?” [11].

For the multivariate analysis, we selected the following subset of these variables based on the current literature, indicators of cardiovascular health and prevalence among Veterans. Older age [12,13] as well as STEMI (versus NSTEMI) diagnosis [14,15] have been associated with later presentation in the general population and were included in our multivariate analysis. Veterans may have to travel longer to reach a VHA facility (versus a local hospital) and studies suggest that distance may influence their care seeking behaviors [16]. For this reason, we felt that distance from the Veteran’s home zip code to the facility was important to include despite approximately 40% missing data. To accommodate distance, we created a categorical variable with a cut point at 50 miles, and an indicator of missing data.

Based on the late presentation literature we included indicators of a history of diabetes [15,17], MI [4], and whether the Veteran experienced one or more episodes of angina daily in the prior four weeks [4] as covariates. The number of angina episodes in the 24 hours prior to hospital admission was also included as a covariate. Like distance, data for the number of angina episodes was missing for a considerable number of patients (30%), however we felt that it was an important indicator of the Veteran’s symptom experience and therefore created a categorical variable with a missing indicator. We also included indicators of a history of CHF [15] or cardiac procedure (including PCI or CABG) [18] to estimate cardiovascular health prior to admission [4]. We included a history of PTSD and a history of alcohol or substance abuse in the multivariate model based on their high prevalence among Veterans and their potential for delaying presentation [19]. Finally, we included a reformulated categorical variable for access to care. We collapsed the 5 categories into 3 (not or not very difficult, somewhat difficult, moderately or extremely difficult) plus a missing indicator to avoid small cell sizes.

### Statistical analysis

Means and standard deviations were calculated for continuous variables and percentages were calculated for categorical variables. Bivariate comparisons were made using independent sample t tests for continuous variables and chi squared tests for categorical variables. Bivariate comparisons between Veterans included in and excluded from our analytic cohort are reported in Table 1 and bivariate comparisons of early and late presenters are reported in Table 2. Independent predictors of late presentation were identified using a multivariate logistic regression model. This model included 13 covariates selected based on the current literature, indicators of cardiovascular health and prevalence among Veterans. Odds ratios (OR) and 95% confidence intervals (CI) from the covariates in the model are reported in Table 3. We evaluated model fit using a Pearson chi-squared goodness of fit test and discrimination using the area under the receiver operating characteristics curve (AUROC). All statistical comparisons use a two-tailed p value <0.05. Statistical analyses were conducted using Stata *Statistical Software: Release 11*. College Station, TX: StataCorp LP.

**Table 2:** Bivariate comparison of demographics,presentation characteristics, medical historyand perception of healthand access to healthcare between early andlate presenters*

	**Early n = 106**	**Late n = 229**	***P***
	***%(n)***	***%(n)***	
**Demographics**			
Age	63.1 ±10.5 (106)	65 ±10.4 (229)	0.12
White	91.4 (96)	85.9 (183)	0.59
Married	47.2 (50)	49.3 (107)	0.89
Lives alone	25.5 (27)	31.4 (72)	0.27
Attended some college/vocational	66 (70)	52.8 (121)	0.02
Miles from home zip to VHA facility	55.5 ±177 (69)	44.6 ±82.7 (130)	0.55
Distance traveled from home residence			0.35
<50 miles	49.1 (52)	42.8 (98)	
≥50 miles	16 (17)	14 (32)	
Missing	34.9 (37)	43.2 (99)	
Employed	30.2 (32)	33.8 (78)	0.52
Family history of CAD	49.1 (52)	43.7 (94)	0.64
**Presentation characteristics**			
STEMI	27.4 (29)	14 (32)	0.00
Angina episodes in previous 24 h			<0.01
0-1	68.9 (73)	24.5 (56)	
≥2	16 (17)	36.7 (84)	
Missing	15.1 (16)	38.9 (89)	
Tertiary facility	92.5 (98)	89.1 (204)	0.34
**Medical history**			
≥1 angina episode per day over prior 4 weeks	17.9 (19)	27.1 (62)	0.07
Cardiac procedure (PCI or CABG)	41.5 (44)	38.4 (88)	0.59
Prior myocardial infarction	42.5 (45)	36.2 (83)	0.28
Prior congestive heart failure	12.3 (13)	17.9 (41)	0.19
Prior stroke	9.4 (10)	14.4 (33)	0.21
Diabetes	40.6 (43)	41.9 (96)	0.82
PTSD	10.4 (11)	13.1 (30)	0.48
Alcohol or substance abuse	21.7 (23)	19.2 (44)	0.60
**Perception of health andhealth care**			
How often do you worry about having a heart attack?			0.83
Never	28.8 (30)	29.8 (64)	
Rarely	26 (27)	29.3 (63)	
Occasionally	28.8 (30)	23.7 (51)	
Often	12.5 (13)	14.4 (31)	
Can't stop	3.8 (4)	2.8 (6)	
How difficult is it for you to get medical care when needed?			0.67
Not a problem	53.8 (57)	48.1 (104)	
Not very difficult	18.9 (20)	25.9 (56)	
Somewhat difficult	11.3 (12)	13.9 (30)	
Moderately difficult	10.4 (11)	5.6 (12)	
Extremely difficult	2.8 (3)	5.1 (11)	

* Late presentation is hospital admission >12 hours after symptom onset of acute myocardial infarction.

*CAD* coronary artery disease; *STEMI* ST elevation myocardial infarction; *PCI* percutaneous coronary intervention; *CABG* cardiac artery bypass graft; *PTSD* post traumatic stress disorder.

**Table 3:** Multivariate logistic regression predictinglate presentation* among Veterans

	**Predictors of late presentation(n = 297)**	***P***
	**OR [95% CI]**	
**Demographics**		
Age ≥65	1.35 [0.72,2.56]	0.35
Lives alone	1.74 [0.9,3.34]	0.1
Distance traveled from home residence		
<50 miles	ref.	
≥50 miles	0.73 [0.31,1.72]	0.47
Missing	1.3 [0.67,2.5]	0.44
Attended some college/vocational	0.68 [0.35,1.33]	0.26
**Presentation**		
STEMI	0.43 [0.2,0.9]	0.03
Angina episodes in previous 24 h		
0-1	ref	
≥2	7.5 [3.6,15.6]	<0.01
Missing	7.43 [3.49,15.84]	<0.01
**Medical history**		
≥1 angina episode per day over prior 4 weeks	1.3 [0.64,2.65]	0.47
Prior cardiac procedure (PCI of CABG)	0.82 [0.4,1.66]	0.57
Prior myocardial infarction	0.55 [0.27,1.11]	0.09
Congestive heart failure	1.64 [0.65,4.14]	0.29
Diabetes	1.09 [0.58,2.05]	0.78
PTSD	2.36 [0.88,6.3]	0.09
Alcohol or substance abuse	0.76 [0.36,1.57]	0.45
**Perception of health care**		
How difficult is it for you to get medical care when needed?		
Not or not very difficult	ref.	
Somewhat difficult	1.16 [0.47,2.88]	0.74
Moderately or more difficult	0.65 [0.28,1.54]	0.33
Missing	0.15 [0.02,1.17]	0.07

* Late presentation is hospital admission >12 hours after symptom onset of acute myocardial infarction.

*STEMI* ST elevation myocardial infarction; *PCI* percutaneous coronary intervention; *CABG* cardiac artery bypass graft; *PTSD* post traumatic stress disorder; OR odds ratio; CI confidence interval.

## Results

**Figure 2:**
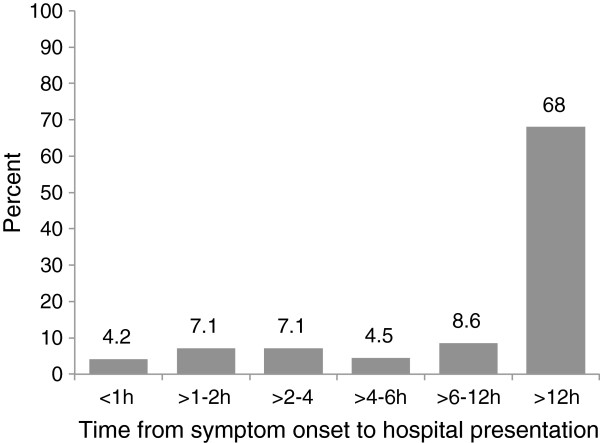
**Time from symptom onsetto presentation among Veteransenrolled in the VHAACS Study.**

The 335 male Veterans that made up our analytic cohort had a mean age of 64 ± 10 years and the majority were white (88%). Forty-nine percent were married and 44% had a family history of coronary artery disease (CAD). A quarter found obtaining healthcare when needed somewhat or more difficult. Twelve percent of these Veterans had a history of PTSD and 20% had a history of alcohol or substance abuse. Forty-two percent had diabetes while 38% had a prior MI and 16% had prior CHF. Thirty one percent of Veterans were missing data on angina in the 24 hours prior to admission and 30% reported ≥2 episodes. As shown in Figure 2, 68% of Veterans presenting to the VHA facilities in the VHA ACS Study with AMI were late presenters.

Late presenters were less likely to have attended some college or vocational training compared to early presenters (late 53% vs. early 66%, p = 0.02) and Veterans having a STEMI were more likely to present early (late 14% vs. early 27%, p = 0.003) (Table 2). Late presenting Veterans were more likely to have ≥2 angina episodes or to be missing data on angina episodes in the 24 hours prior to admission (≥2 episodes, late 38% vs. early 16%, p < 0.01 and missing data late 39% vs. early 15%, p < 0.01) (Table 2).

We used multivariate logistic regression to adjust for covariates (described in Methods) to identify independent predictors of late presentation. We found that when adjusting for other variables, having a STEMI significantly protected against late presentation (OR = 0.4 95%CI [0.2, 0.9]). In addition, we found that having ≥2 angina episodes or missing data on angina in the 24 hours prior to hospital admission (versus having 0-1 angina episodes) significantly predicted late presentation (≥2 angina episodes OR = 7.5, 95%CI[3.6, 15.6] as did missing data OR = 7.4 95%CI[3.5, 15.8]). This model had good fit and moderate discrimination (Pearson goodness of fit statistic p > 0.05 and AUROC = 0.8).

## Discussion

In summary, we did not find many significant differences between late and early presenting Veterans. Veterans attending some college or vocational school were more likely to present early, however this effect did not hold when adjusting for other variables. Having a STEMI was independently associated with presenting early and reporting ≥2 angina episodes or missing data for the 24 hours prior to hospital admission were independently associated with presenting late. Overall, our findings were both similar and different from what has been found to predict late presentation in the general population.

Our finding that Veterans having a STEMI (vs. NSTEMI) were more likely to present early is consistent with what has been found in the general population [7,15]. Ting et al found that STEMI patients with the shortest delay times had the highest adjusted in-hospital mortality rates [7,17]. These findings suggest that the shortest delays may be an indicator of more severe symptoms and therefore more severe underlying disease. More generally, longer delays are associated with a decreased likelihood for reperfusion therapy and higher mortality rates in STEMI patients [7]. For NSTEMI patients, longer delays have not been associated with increased mortality [15]. This is encouraging given that 82% of our analytic cohort had an NSTEMI diagnosis. However, because neither patients nor physicians can reliably differentiate between STEMI and NSTEMI without an ECG, and symptoms can manifest very differently for different patients, efforts to decrease delay should be taken for all patients with potential AMI [15,17].

We also found that Veterans having ≥2 angina episodes or missing data on angina episodes in the 24 hours prior to admission were significantly more likely to present late. This finding is somewhat difficult to interpret given the missing data. For those patients with complete data, having more angina episodes in the previous 24 hours may simply reflect that they had been having the same symptoms for longer. The association of missingness and late presentation may in part be based on the self reported nature of the variable. Veterans who have waited longer to present may be less likely to remember the frequency or duration of their symptoms.

There were several factors associated with late presentation in the general population that were not associated with late presentation among Veterans. These included comorbid conditions and living alone. In a study comparing Veterans with AMI to their Medicare counterparts, Peterson et al. found that Veterans had a higher prevalence of many comorbidities including diabetes and stroke [10]. While comorbid conditions have been associated with atypical symptoms of AMI leading to increased delays in studies in the general population [4], we did not find such relationships despite Veterans’ higher prevalence of comorbidities. This may be associated with our initial exclusions of Veterans with concurrent noncardiac admission or DNR. In addition, we were somewhat limited in the number of covariates we could include in the model and were therefore not able to examine all potential comorbidities.

Living alone or being single (versus married) has been associated with delayed presentation in the general population especially among men [20,21]. This is of particular concern among Veterans who are predominantly male and may be at a higher risk of living alone due to difficulties forming social attachments or PTSD based on combat experiences [22]. Other physical and mental vestiges of military service such as physical disability or substance abuse, may further contribute to self-isolation among Veterans. An estimated quarter to a third of VHA users live alone [23]. Despite these concerns, we did not find an association of living alone and late presentation among Veterans. This may be explained in part by findings reported by Guzman et al. that older Veterans who live alone actually have increased outpatient service utilization suggesting that Veterans living alone may seek out social support through health care encounters [23]. This finding of increased outpatient service utilization suggests both a risk and a benefit for late presentation among Veterans. The risk is that Veterans living alone may contact or present to their primary care doctor before going to the hospital, further delaying presentation. The benefit however is that more frequent healthcare encounters creates the opportunity for targeted patient education for those at high risk of AMI.

While patient education efforts have had limited success in the general population [24,25], the 2004 VHA patient education intervention known as *Time is Life* (TiL) suggests the potential for success among Veterans. The TiL intervention asked providers to distribute educational materials to high risk Veterans during outpatient services. The educational materials promoted development of a survival plan, early recognition of symptoms and calling an ambulance at the first sign of AMI. Of the 4,884 Veterans surveyed, 2,593 responded. Eighty-two percent of respondents said that they never saw the patient education materials. After reviewing the materials, 90% responded that they would call an ambulance if they experienced symptoms of AMI. *Personal communication with programdirector Sandra Pineros ofthe VA Puget Sound’sHealth Care System coordinatingcenter, regarding the unpublishedshort communication “An evaluationof a patient educationinitiative in VHA: Timeis Life for HeartAttack”]* Thus, there is evidence that Veterans would be receptive to a targeted patient education intervention. The TiL study also illuminates a system failure to disseminate patient materials during outpatient visits. Future patient education interventions to decrease late presentation among Veterans must have effective strategies in place to avoid such system failures. Focused efforts on dissemination and provider buy-in may help avoid this pitfall with future interventions.

### Limitations

A major limitation of our study is that our research questions and analysis were designed post hoc. In addition, we excluded patients based on several criteria including having a concurrent acute noncardiac condition at admission biasing our cohort towards much healthier patients. The majority of those excluded were early presenters which may have also biased our analysis. In this case, early presentation may be associated with the noncardiac condition for which they were admitted. Participants missing time from symptom onset may have not been able to report or remember the time of symptom onset (based on poor health or length of elapsed time), thus excluding them may have also introduced significant bias. Nearly half of the Veterans in our study were missing information on angina within the prior 24 hours, which may have been a significant predictor of delay if we had complete data. Those participants missing this data were significantly more likely to be late presenters suggesting potential unmeasured confounding. Finally, the self-report nature of our outcome measure may have further been subject to patient recall bias.

## Conclusion

Sixty-eight percent of Veterans presenting to VHA facilities with AMI are late presenters. We found few predictors of late presentation. In bivariate comparisons, attending some college or vocational school was associated with early presentation. In multivariate analysis adjusting for other predictors of late presentation, having a STEMI was independently associated with coming in early and reporting ≥2 angina episodes in the 24 hours prior to hospital admission was independently associated with coming in late. These predictors of late and early presentation are not unlike those found in the general population; however, Veteran’s may have additional opportunities for interventions focused on patient education if the VHA can overcome previous system failures.

## Abbreviations

ACS: Acute coronary syndrome; AMI: Acute myocardial infarction; CABG: Coronary artery bypass grafting; CAD: Coronary artery disease; CHF: Congestive heart failure; CI: Confidence interval; CKD: Chronic kidney disease; COPD: Chronic obstructive pulmonary disease; DNR: Do not resuscitate; ECG: Electrocardiogram; NSTEMI: Non-ST elevated myocardial infarction; OR: Odds ratio; PCI: Percutaneous coronary intervention; PTSD: Post traumatic stress disorder; STEMI: ST elevated myocardial infarction; VHA: Veterans Health Administration.

## Competing interests

The authors declare that they have no competing interests.

## Author’s contributions

KM and CM designed and completed the analysis. CM, RT EL and SF all provided critical feedback during the conceptual phase of the analysis. KM drafted the initial manuscript. CM, RT, EL, and SF all revised and approved the final manuscript.

## Pre-publication history

The pre-publication history for this paper can be accessed here:

http://www.biomedcentral.com/1471-2261/12/82/prepub
